# ^99m^Tc Pyrene Derivative Complex Causes Double-Strand Breaks in dsDNA Mainly through Cluster-Mediated Indirect Effect in Aqueous Solution

**DOI:** 10.1371/journal.pone.0108162

**Published:** 2014-09-22

**Authors:** Wei-Ju Chung, Yujia Cui, Feng-Yun J. Huang, Tzu-Hui Tu, Tzu-Sen Yang, Jem-Mau Lo, Chi-Shiun Chiang, Ian C. Hsu

**Affiliations:** 1 Department of Biomedical Engineering and Environmental Sciences, National Tsing Hua University, Hsinchu, Taiwan; 2 School of Dental Technology, College of Oral Medicine, Taipei Medical University, Taipei, Taiwan; St. Georges University of London, United Kingdom

## Abstract

Radiation therapy for cancer patients works by ionizing damage to nuclear DNA, primarily by creating double-strand breaks (DSB). A major shortcoming of traditional radiation therapy is the set of side effect associated with its long-range interaction with nearby tissues. Low-energy Auger electrons have the advantage of an extremely short effective range, minimizing damage to healthy tissue. Consequently, the isotope ^99m^Tc, an Auger electron source, is currently being studied for its beneficial potential in cancer treatment. We examined the dose effect of a pyrene derivative ^99m^Tc complex on plasmid DNA by using gel electrophoresis in both aqueous and methanol solutions. In aqueous solutions, the average yield per decay for double-strand breaks is 0.011±0.005 at low dose range, decreasing to 0.0005±0.0003 in the presence of 1 M dimethyl sulfoxide (DMSO). The apparent yield per decay for single-strand breaks (SSB) is 0.04±0.02, decreasing to approximately a fifth with 1 M DMSO. In methanol, the average yield per decay of DSB is 0.54±0.06 and drops to undetectable levels in 2 M DMSO. The SSB yield per decay is 7.2±0.2, changing to 0.4±0.2 in the presence of 2 M DMSO. The 95% decrease in the yield of DSB in DMSO indicates that the main mechanism for DSB formation is through indirect effect, possibly by cooperative binding or clustering of intercalators. In the presence of non-radioactive ligands at a near saturation concentration, where radioactive Tc compounds do not form large clusters, the yield of SSB stays the same while the yield of DSB decreases to the value in DMSO. DSBs generated by ^99m^Tc conjugated to intercalators are primarily caused by indirect effects through clustering.

## Introduction

Radiation therapy is a vital tool in cancer treatment, used either in isolation or tandem with surgery or chemotherapy. It involves the emission of charged particles that generate double-strand breaks in nuclear DNA, leading to cell apoptosis. However, this process tends to be indiscriminate, damaging healthy tissues and causing unwanted side effects. New radiation sources and accurate dosage measurements are research areas that can lead to improved treatment for cancer patients.

Among radioactive sources, Auger electron emitters demonstrate the advantage of a short effective range (in nm), which helps minimize tissue damage if delivered directly to the nucleus of cancerous cells. The majority of Auger electrons have less than 1 keV of energy and are often emitted in showers of tens of electrons per decay. Auger electron emission is a high linear energy transfer (LET) process that creates high ionization density and can cause complex clustered DNA damage (two or more lesions within one or two helical turns) and isolated lesions along the tracks of the Auger electrons through direct ionization (direct effect) and the interactions between DNA and hydroxyl free radicals generated in the solution (indirect effect). The lesions include DSBs and non DSBs like SSBs, oxidized bases and abasic sites [1], [2], [3]. Clustered lesions are more difficult for cells to repair than isolated lesions, and often result in additional DSBs and high mutation rates close to the site post radiation. For this reason, clustered lesions are considered highly toxic [4].

^99m^Tc, which has been studied as a potential Auger electron donor, produces 4 Auger electrons per decay and has a short half-life (6.01 h), making it less toxic than any isotope used in conventional radiation therapy [5]. These electrons are highly effective at generating DSBs in nuclear DNA, and various conjugates are being investigated as potential candidates for cancer treatment [6], [7], [8], [9], [10], [11]. Several studies have attempted to characterize ^99m^Tc and its interactions with double-strand DNA (dsDNA). Simulations [12], [13] have shown that if this nuclide is placed within DNA base pairs, it generates an average of 0.4–0.8 DSBs per decay. Experiments performed in bulk using gel electrophoresis have indicated that if ^99m^Tc is conjugated with the DNA intercalator N-(2-amino-ethyl)-N′-pyrene-l-ylmethyl-ethane-1,2-diamine (APMED) to form ^99m^Tc-APMED, it induces double-strand breaks in plasmid DNA, although at a much lower yield (0.005) than predicted [5]. This is partly because the Tc atom is a few angstroms away from the DNA backbone [6] while the nuclide is placed on the bases in simulations. Recent studies on ^125^I-labeled Hoechst derivatives have shown that a critical distance exists between the ^125^I atom and the DNA helix, where DSB production switches from direct to indirect mechanisms [14]. The topology and local concentration of DNA molecules also affects how DSBs form [15]. Under crowded conditions, free radicals from nearby Auger electrons on different DNA molecules can cause some DSBs through multiple SSBs, resulting in additional linear and open circular DNA forms.

A trifunctional complex that has a nucleus targeting sequence attached to ^99m^Tc-APMED, targeting and accumulating in the nucleus of B16F1 mouse melanoma cells, was shown to have a much stronger radiotoxic effect than that of nonnucleus-localizing ^99m^Tc complexes. This is possibly due to DSBs occurring in the cell nucleus [6].

Despite recent advances in the synthesis of ^99m^Tc complexes and in vivo demonstrations of their effectiveness as a cell apoptosis agent, the mechanism of DNA-^99m^Tc complex interactions has not been studied in depth. Also, the conformations of plasmids are different in buffered aqueous solution (B-form) and in methanol (P-form) [16]. In this study, we examined the effect of the ^99m^Tc on plasmid DNA in dosimetry by using gel electrophoresis in both aqueous and methanol solutions. Because DMSO reduces the DNA damage by free radicals [17], various amounts of DMSO were added to further distinguish between the direct and indirect effects of causing strand breaks.

## Materials and Methods

All chemicals and solvents were purchased and used as is. N-(2-aminoethyl)-N′-pyrene-1-ylmethylethane-1,2-diamine (APMED) compound were prepared according to Häfliger, P., et al [5].

### Preparation of [^99m^Tc (CO)_3_(OH_2_)_3_] ^+^ solution

Sodium carbonate (4 mg, 0.037 mmol, Riedel-deHaën), sodium borohydride (6 mg, 0.159 mmol, Acros Organics), and di-sodium tartratedihydrate (20 mg, 0.087 mmol, MERCK) were loaded into an injection vial and sealed with an aluminum foil wrapped rubber stopper. The sealed vial was filled with 1 atm CO for 10 min and a balloon filled with CO was connected to the vial through the rubber stopper. Na^99m^TcO_4_ was eluted from a Technetium (^99m^Tc) generator (iba, Elumatic III) in 0.9% saline and 1 ml of the eluted solution was injected into the vial by syringe. The solution was shaken at 80 rpm and heated at 75°C in a water bath for 30 min. After the reaction was completed, the pH value of solution was adjusted to 7 by adding 2-N-morpholinoethonesulfonic-HCl buffer.

### Preparation of ^99m^Tc complex with APMED (^99m^Tc-APMED)

After adding 10^−3^ M aqueous solution (0.03 ml) of APMED into the tricarbonyl ^99m^Tc solution vial from the previous step, the vial was heated to 98°C for 40 min and then cooled to room temperature. The entire mixture was purified with an HPLC system (Waters Sunfire C18, 19×150 mm). The flow rate was 4 ml/min, with the mobile phases were 0.1% trifluoroacetic acid (solvent A) and HPLC-grade MeOH (solvent B). Gradient: 0–3 min: 100% A; 3–8 min: 75% A, 25% B; 8–30 min: 45–0% A, 55–100% B; 30–33 min: 100% B; 33–35 min: 100% A. Fractions with radioactivity were collected and dried. The resulting ^99m^Tc-APMED was dissolved in either TE buffer with 4% Tween 80 (Sigma-Aldrich) for use in aqueous solutions or in 100% methanol.

### Preparation of pIRES plasmid DNA

Supercoiled plasmid DNA pIRES (6.1 kbp, Clontech Laboratories, Inc.) was transformed into E. coli DH5α competent cells (Food Industry Research and Development Institute (FIRDI), Taiwan) and amplified. After purification with FaverPrep Plasmid DNA Extraction Mini Kit (Favorgen Biotech Corp.), the plasmid DNA was stored in 10 mM Tris, pH 8, 1 mM EDTA (TE buffer) at 4°C and used within a week. Linear pIRES was prepared by digesting the supercoiled plasmids with Xho I, which creates a single DSB. Relaxed circular form pIRES was prepared by digestion with N.Alw1 (New England Biolab) without further purification.

### Gel electrophoresis analysis of strand breaks caused by ^99m^Tc-APMED

For experiments in aqueous solutions, ^99m^Tc-APMED was dissolved in TE buffer with 4% Tween 80 (Sigma-Aldrich), with and without DMSO, and then incubated in total darkness with pIRES DNA for 12 or 24 h at 4°C.

For experiments under saturation conditions, concentrated APMED was mixed with dissolved ^99m^Tc-APMED before incubation with 100 ng pIRES plasmids. The total concentration of APMED and Tc-APMED was 3 µM during incubation.

For experiments done in methanol, ^99m^Tc-APMED was dissolved in 100% methanol. 52.5 µl of ^99m^Tc-APMED solution was mixed with 10 µl DNA (0.35 µg) and incubated at room temperature for 3, 6, 12 or 24 h. Afterwards, the solutions were dried and reconstituted with 28 µl 50 mM Tris, pH 7.4 (Tris buffer) before loaded onto gels. The samples with DMSO were incubated from 23 to 37 h.

100 ng pIRES DNA was loaded in each lane in 1% agarose gel in 1x TAE, running at 8.3 V/cm for 60 min. The gels were stained with EtBr (0.1 µg/ml) for 2 h, and destained for 10–30 min. The images were analyzed by Image J (NIH). Samples incubated with saturating amount of APMED were run with 0.2 µg/ml ethidium bromide in the gel and running buffer instead and destained for 1.5–2 h.

The fraction of each type of DNA was calculated from individual lanes without taking into account invisible fragmented DNA. This ensures that any DNA loading errors would not affect our data. Since ethidium bromide stains supercoiled DNA and linear or relaxed DNA with different intensities, a correction factor of 1.45 [18] was used for the intensity of supercoiled DNA bands.

Assuming ^99m^Tc-APMED binds to dsDNA according to a Poisson distribution, the mean number of SSBs per molecule (X_SSB_) and the mean number of DSBs per molecule (X_DSB_) can be calculated from the fraction of supercoiled DNA remained (F_sc_) and the fraction of linear DNA formed (F_L_) after irradiation by ^99m^Tc-APMED [19]:
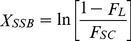
(1)

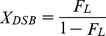
(2)


The average number of DSB or SSB as a result of accumulated decays can be obtained by plotting X_DSB_ or X_SSB_ against total accumulated decays per molecule and calculating the slope. The yield of DSB or SSB per decay can be calculated as:
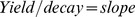
(3)


This method takes into account any pre-existing DSBs or SSBs. The total number of ^99m^Tc actually bound to the DNA molecules was estimated using the binding constant of this compound [5], and the ratio of ^99m^Tc-APMED to total Tc-APMED can be deduced from the time of the last elusion from the Tc generator and the time of the experiment.

The theoretical calculations regarding DSB include two parts. The first is the contribution from “nickers” (mostly indirect effect of Auger electrons), assuming that SSBs on the DNA molecules follow Poisson distribution and that two closely spaced SSB result in a DSB. The second part is the contribution from “cutters”, or direct effect.

## Results and Discussion

### DNA strand breaks by ^99m^Tc-APMED in agarose gels

Gel electrophoresis is a useful tool in examining strand breaks. Supercoiled DNA transforms into relaxed circular DNA when it undergoes a SSB, and linear in the presence of DSBs or multiple SSBs/DSBs near each other. After incubation of ^99m^Tc-APMED with plasmid DNA, different forms of DNA form distinct bands, allowing the change in the fraction of each form to be calculated from the fluorescent intensity of the bands. To minimize DNA sample degradation over time, freshly prepared pIRES plasmids were used for all experiments and the incubation with ^99m^Tc was done at 4°C. Since Tris is considered a hydroxyl scavenger for long range radiation studies such as γ-radiation, controls were done to compare the yields in Phosphate buffer (10 mM Phosphate, 1mM EDTA, pH 8.0) and Tris buffer (10 mM Tris, 1 mM EDTA, pH 8.0) side by side on same gels. Both SSB and DSB yields are comparable. It is not clear from this data alone whether Tris is more effective in scavenging hydroxyls.

Incubations in buffered aqueous solutions were conducted both in the absence and presence of the free-radical scavenger DMSO [17]. In the absence of DMSO, the yields of DSB and SSB are the results of both direct and indirect effects caused by Auger electrons. In the presence of DMSO, DSBs and SSBs are the results of direct interactions of Auger electrons with the DNA backbone, and not the results of free radicals generated by Auger electrons.

Due to the short half-life of ^99m^Tc, different doses are obtained by adjusting the molar ratio of ^99m^Tc-APMED molecules to DNA molecules. The volume of all samples in each gel was the same, as were the length of incubation. Therefore all data points from the same gel share the same decay to total Tc-APMED ratio. This is different from Auger electron emitters with longer half-lives. The half-life of ^125^I is about 60 days. Hence, all accumulated decays are from the same ^125^I-to-DNA ratio obtained at various times. There is no noticeable difference in strand break yield per decay between the results from 12 h and 24 h incubation times for ^99m^Tc at low doses.

**Figure 1:**
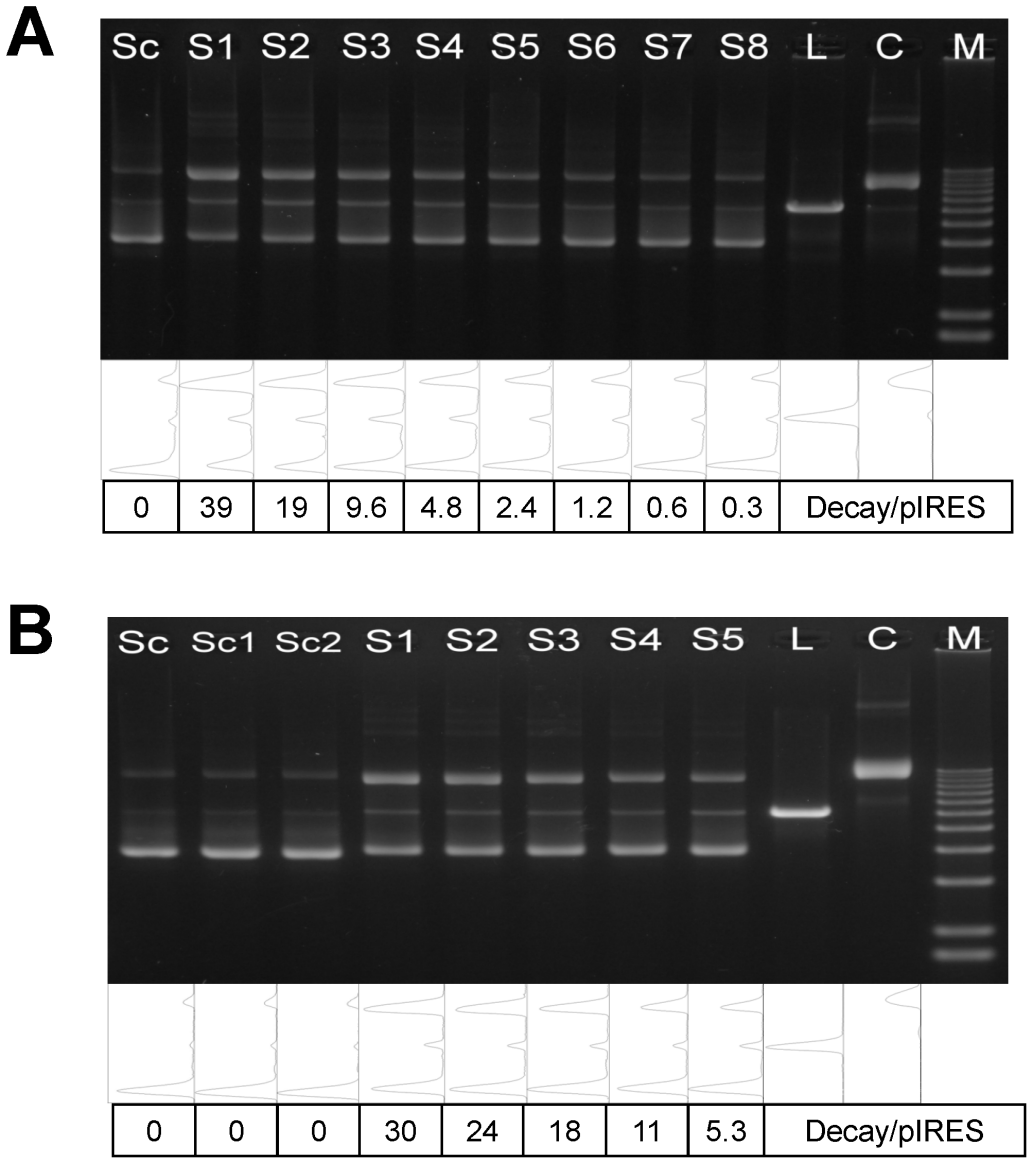
Agarose gel electrophoresis of supercoiled plasmid pIRES incubated with various radioactivity doses. Supercoiled plasmid pIRES (6.1 kbp) incubated with different doses of ^99m^Tc-APMED in 10 mM Tris, 1 mM EDTA, pH 8.0 (TE buffer) with 4% Tween 80 for 12 h at 4°C. 100 ng DNA was loaded into each lane. The line scan and doses (Decay/pIRES) of each lane are presented below the gels. Sc: supercoiled plasmid; L: linear DNA; C: relaxed circular DNA; M: DNA marker. (A) Without DMSO. The plasmids in Lane Sc, L, and C were not irradiated. (B) With 5 mM DMSO. Sc, Sc1, and Sc2 were control lanes. Sc: TE buffer only; Sc1: with 4% Tween 80; Sc2, L, C: with 4% Tween 80 and 5 mM DMSO.

Figure 1A shows that incubation with ^99m^Tc-APMED causes both SSBs and DSBs in the absence of DMSO. The fraction of linear DNA remains small, even when over half of the supercoils have unwound, implying that the yield of DSB is much lower than that of SSB. In contrast to the results from ^125^I studies, in which SSBs disappear with the addition of DMSO, the presence of DMSO does not considerably hinder the formation of SSBs from ^99m^Tc-APMED, as shown in Figure 1B.

### Quantification of strand break yield in buffered aqueous solution

**Figure 2:**
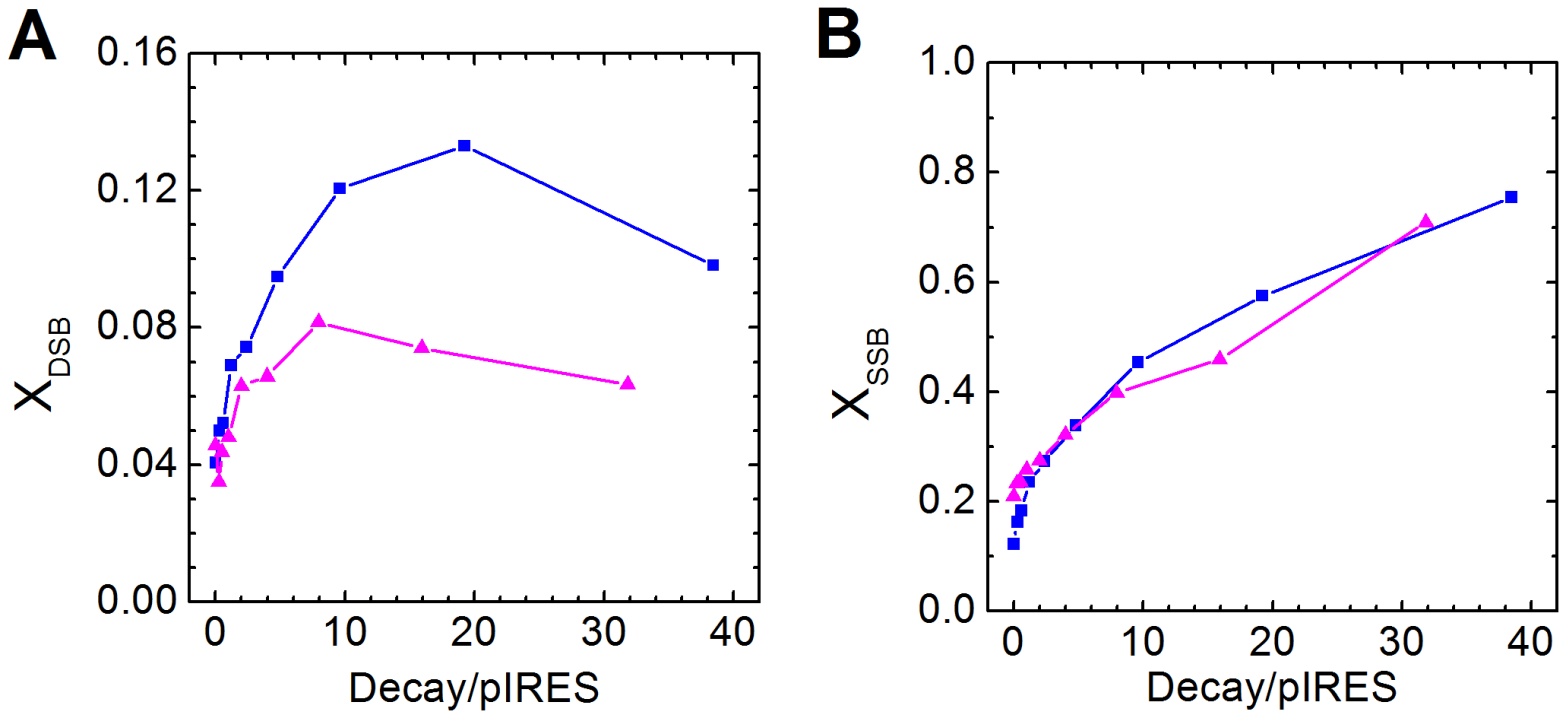
Quantification of average strand breaks per DNA molecule without DMSO. There were two independent experiments (blue ▪ and magenta ▴). (A) The mean number of double-strand breaks per DNA molecule (X_DSB_) to accumulated decays per plasmid. (B) The mean number of single-strand breaks per DNA molecule (X_SSB_) to accumulated decays per plasmid.

The fractions of each type of DNA can be calculated using gels such as those shown in Figure 1. Based on the fractions of linear DNA and supercoiled DNA, the mean number of DSB and SSB per DNA molecule (X_DSB_ and X_SSB_ respectively) can be deduced using equations 1 and 2, assuming ^99m^Tc-APMED binds to plasmids randomly, so that the distribution of SSBs follows Poisson distribution. Figure 2 shows the results from two independent experiments. For results of the duplicates, please see Figure S1 and Table S1. At low doses (<6 decays/plasmid), both X_DSB_ and X_SSB_ increase linearly with the total number of decays per plasmid, as predicted [19]. In the presence of DMSO, as shown in Figure 1B, DSB yield decreases to about one twentieth of the previous value. This is a strong indicator that for ^99m^Tc-APMED, the main contribution of DSB comes from multiple neighboring SSBs.

If SSBs follow Poisson distribution, in any case, the fraction of DSBs as the result of multiple SSBs close together cannot be bigger than the sum of all fractions of plasmids having two or more SSBs. In fact, it should be much smaller.

For example, when X_SSB_ is 0.3, assuming it is all from radiation, the sum of all fractions with two or more SSBs is 0.037, according to the relevant Poisson distribution. However, the fraction of linear DNA after adjusting for the fraction present before irradiation using data from Figure 2A is 0.05, which is significantly higher than expected. The only way to produce that level of increase in the fraction of linear DNA with the observed increase of SSBs seen in the gels is for the SSBs to not follow a Poisson distribution. Instead of random occurrences, the SSBs need to cluster together to account for the elevated level of X_DSB_, and so equation 1 in this case predicts the behavior of clusters instead of single SSBs. We therefore call the yield calculated this way the apparent yield of SSB.

Tc-APMED is a DNA intercalator. Over 99% of all ligands were bound to DNA at the applied molar ratio, which was substantially below the saturation point for Tc-APMED. The cooperative binding of DNA intercalators [20], in which the binding of one intercalator slightly lowers the energy barrier of binding to nearby sites, thus creating a local cluster of Tc-APMED.

At high doses (>10 decays/plasmid) the apparent SSB yield is consistently around 0.01/decay, which is much smaller than the 0.04/decay at low doses. This could be the result of a combination of larger clusters and more clusters when the ratio of Tc-APMED to plasmid increases. DSB yield also decreases in this range and sometimes even appears to be negative, i.e. X_DSB_ of linear DNA increase slower or decrease as more decays accumulate.

At lower doses, most plasmids have either zero or one cluster. Higher doses have the effect of increasing the number of plasmids that have one cluster and/or the cluster size. At higher doses, the fraction of plasmids with two or more clusters start to increase, which effectively doubles or triples the possibility of having DSBs and eventually leading to two or more DSB on the same molecule and contribute to the decrease of linear DNA fraction.

Although the increase in cluster size does not change the ratio of SSB to the number of Tc-APMED in the cluster, it does increase the number of SSB per cluster. For a given ratio of SSB to number of Tc-APMED, assuming SSBs follow Poisson distribution within each cluster, the number of clusters that produce at least one DSB increases with cluster size, as shown through simulations. The condition for DSB formation is that two SSBs are generated on opposite strands within 12 bp. The fraction of fragments generated can be estimated through simulations as well by counting fractions that have two DSBs that are at least 100 bp apart. At around 6 decays/molecule, the fragment fraction starts to increase linearly with dose. Combining the two, at around 10 decays/molecule, depending on the ratio of SSB to Tc-APMED, the slope of X_DSB_ vs dose either arise slower, flatten out or decrease.

**Figure 3:**
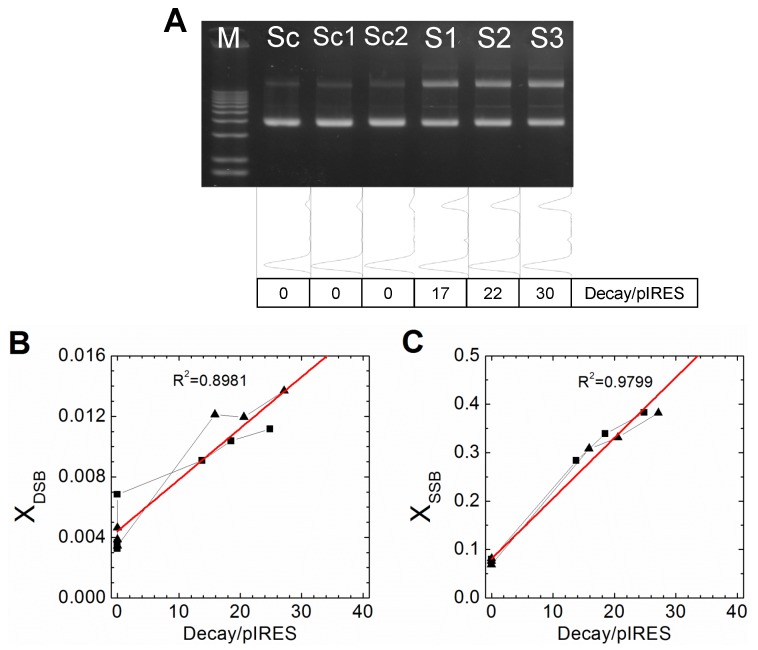
Decay yield of Tc-APMED on supercoiled pIRES plasmids under saturation conditions. Supercoiled plasmid incubated with different doses of ^99m^Tc-APMED and APMED in TE buffer with 4% Tween 80 for 24 h at 4°C. For different doses of ^99m^Tc-APMED, the total concentration of pyrene is kept at 3 µM. The line scan and doses (Decay/pIRES) of each lane are presented below the gel. Sc: supercoiled plasmid; M: DNA marker. Two independent experiments (▪ and ▴) are shown in (B) and (C). (A) Without DMSO. The plasmids in Lane Sc, Sc1, and Sc2 were not irradiated. Sc: TE buffer only; Sc1: with 4% Tween 80; Sc2: with 4% Tween 80 and 3 µM APMED. (B) The mean double-strand breaks per DNA molecule (X_DSB_). The yield of DSB per decay is 0.0003±0.0001 (mean ± SD). (C) The mean single-strand break per DNA molecule (X_SSB_). The SSB yield per decay is 0.0123±0.0004 (mean ± SD). The linear fittings in (B) and (C) were calculated with all data points.

In order to test the hypothesis that Tc-APMED binds in clusters at higher ligand to DNA ratios, experiments were done under near saturation conditions of ligands. For each sample, the amount of APMED was adjusted so that the total concentration of pyrene is 3 µM, making the ratio of ligand/base pair 1∶5. According to calculations based on the binding affinity of APMED to linear DNA, slightly under 92% of all ligands are bound. This arrangement should effectively breakup the proposed clusters of Tc-APMED and therefore decrease the yield of DSB. See Figure 3. The yield of DSB indeed decreased dramatically from 0.011±0.005 under non-saturating conditions to 0.0003±0.0001 and remains unchanged up to 100 mM DMSO. See Table 1. So the main mechanism for DSB formation here is through direct effect when ^99m^Tc-APMED compounds bind to DNA according to a Poisson distribution.

**Table 1:** Strand break yield per decay by ^99m^Tc-APMED in aqueous solution.

[DMSO]	Non-saturation		Saturation	
	DSB/decay	SSB/decay	DSB/decay	SSB/decay
0 mM	0.011±0.005	0.04±0.02	0.0003±0.0001	0.0123±0.0004
5 mM	0.0007±0.0004	0.017±0.001	0.0005±0.0003	0.016±0.005
100 mM	0.0004±0.0001#	0.008±0.001#	0.0003±0.00003	0.007±0.002
1 M	0.0005±0.0003#	0.008±0.001#	NA*	NA*

The data here is given in the form of Value ± SD.

The data were obtained across two independent experiments.

# One independent experiment was performed, and the errors are the fitting errors of the slopes of X_SSB_ and X_DSB_, respectively.

* Not available.

The yield of SSB actually does not increase compared to non-saturation experiments. This contradicts with the idea of more even distribution of ^99m^Tc-APMED, therefore more frequent SSBs in saturation conditions. The reason could be we used APMED instead of non-radioactive Tc-APMED to saturate the plasmids.

The effect of DMSO on ^99m^Tc-APMED seems to be the opposite of that on ^125^I [21] or ^99m^Tc when placed near DNA bases. In the absence of DMSO, the yield of DSB and SSB are approximately 1/100th of that predicted for ^99m^Tc placed in bases, which is consistent with previous findings (Table 1) [5]. This is probably due to the short distance between ^99m^Tc and the DNA backbone. In the presence of 5 mM DMSO, the yield of DSB decreased to one-twentieth of the DMSO-free experiments, which is close to the detection limit of this method. The yield stayed the same when higher DMSO concentrations were used.

In aqueous solutions, DSBs are primarily caused by indirect effects. Although the yield of SSB decreases with the concentration of DMSO, it stabilizes at approximately a quarter of the yield without DMSO. This is consistent with findings regarding ^125^I Hoechst derivatives: when a ^125^I atom is placed beyond a critical distance from the DNA helix, DSBs are produced by indirect effects.

The clustering effect of this Tc-pyrene conjugate makes it a good candidate for in vivo applications, as demonstrated by Haefliger et al [6]. Future cancer drug design can take advantage of the clustering of high affinity DNA intercalators and use them as carriers for Auger electron emitters to enhance toxicity.

### Quantification of strand break yield in methanol

In order to observe indirect effects on a different structural form of DNA, plasmid DNA was incubated with ^99m^Tc-APMED in 85% methanol. The yield of SSB in methanol is 7.2±0.2 per decay, decreasing with DMSO concentration (Table 2). The number is still considerably higher than zero, even in 2 M DMSO. dsDNA assumes P-form in this solution [16], with bases pointed outward and backbones intertwined around each other. As a DNA intercalator, ^99m^Tc-APMED could still bind to the bases by stacking in the midst of or at the ends of DNA molecules [22], or in an orientation distinct from those bound to DNA in aqueous solutions. Because the number of DSB in 85% methanol increased by a factor of 100 compared to those in aqueous solution, it is highly likely that the ^99m^Tc-APMED was situated extremely close to the DNA backbones. In the presence of DMSO, the DSB yield decreases and cannot be detected in 2 M DMSO. DSBs in methanol with P-form DNA, like those in an aqueous solution, are caused by indirect effects.

**Table 2:** Strand break yield per decay by ^99m^Tc-APMED in methanol.

[DMSO]	0 mM&	2 mM$	200 mM$	2 M$
DSB/decay	0.54±0.06	0.04±0.004	0.007±0.002	ND*
SSB/decay	7.2±0.2	0.7±0.1	0.6±0.1	0.4±0.2

The data here is given in the form of Value ± SD.

& The data were obtained across two independent experiments.

$ The data were obtained across three independent experiments.

* Not detectable.

## Conclusions

Our gel electrophoresis-based quantitative analysis has shown that pyrene derivative-conjugated ^99m^Tc causes DSBs by creating closely spaced SSBs on opposite strands of dsDNA through clustering of intecalating ligands. The diminished number of DSBs, but not SSBs, in the presence of high concentrations of the free radical scavenger DMSO suggests that SSBs are caused by both direct and indirect effects, whereas DSBs are caused almost entirely by indirect effects.

## Supporting Information

Figure S1 **Various plots of average strand breaks per DNA molecule against different dosage units.** The colors (blue and magenta) denote two independent experiments. The dashed lines represent duplicates. (A) Average double strand breaks per DNA molecule (X_DSB_) to accumulated decays per ml. (B) Average single strand breaks per DNA molecule (X_SSB_) to accumulated decays per ml. (C) Average double strand breaks (X_DSB_) to accumulated decays per plasmid. (D) Average single strand breaks (X_SSB_) to accumulated decays per plasmid. The low dosage data shown in (E) and (F) were used to calculate the respective yields.
(TIF)

Table S1 **Strand break yield per decay by ^99m^Tc-APMED in aqueous solution without DMSO.**
(DOC)
