# Supplementary material for: 99mTc Pyrene Derivative Complex Causes Double-Strand Breaks in dsDNA Mainly through Cluster-Mediated Indirect Effect in Aqueous Solution
Source: PLoS One. 2014 Sep 22;9(9):e108162. doi: 10.1371/journal.pone.0108162 (PMC4171534; doi:10.1371/journal.pone.0108162)
Supplement: Table S1 — Strand break yield per decay by 99mTc-APMED in aqueous solution without DMSO. (DOC) [file pone.0108162.s002.doc]

**Table S1.** Strand break yield per decay by ^99m^Tc-APMED in aqueous solution without DMSO.

| Experiment | 1 | | 2 | |
| --- | --- | --- | --- | --- |
| Apparent SSB yield/decay | 0.026 | 0.023 | 0.061 | 0.063 |
| DSB yield/decay | 0.007 | 0.007 | 0.014 | 0.017 |

There was a duplication for each experiment.
